# Danish translation and cultural adaption of the Person-Centred Practice Inventory-Staff and Person-Centred Practice Inventory-Care questionnaires

**DOI:** 10.3389/frhs.2025.1559443

**Published:** 2025-07-07

**Authors:** Elizabeth Rosted, Mette Kjerholt, Bibi Hølge-Hazelton, Tanya McCance, Brendan McCormack, Thora Thomsen

**Affiliations:** ^1^ Department of Oncology and Palliative Care, Zealand University Hospital, Roskilde, Denmark; ^2^ Department of Regional Health Research, University of Southern Denmark, Odense, Denmark; ^3^ Department of Hematology, Zealand University Hospital, Roskilde, Denmark; ^4^ Research Support Unit, Zealand University Hospital, Roskilde, Denmark; ^5^ Faculty of Life and Health Sciences, School of Nursing and Paramedic Science/Institute of Nursing and Health Research, Ulster University, Coleraine, United Kingdom; ^6^ Faculty of Medicine and Health, The University of Sydney, Sydney, NSW, Australia; ^7^ Department of Ear, Nose, Throat and Maxillofacial Surgery, Zealand University Hospital, Køge, Denmark

**Keywords:** translation, person-centred practice, cross-cultural adaptation, person-centred, measurement

## Abstract

**Background:**

Internationally, person-centred practice is a recognized standard of quality care influencing the experience of care for healthcare professionals, service users, families and care partners. To measure the experience from the perspectives of both caregivers and patients, the instruments Person-Centred Practice Inventory-Staff (PCPI-S) and the Person-Centred Practice Inventory-Care (PCPI-C) have been developed, which are both theoretically aligned with McCormack and McCance's person-centred framework. In this paper, we present translation and cultural adaption of the questionnaires into Danish.

**Methods:**

A model including translation and cultural adaption of both the PCPI-S and the PCPI-C questionnaires was used. The translation and cultural adaption took place from September 2021 to March 2022 and was conducted within the context of a Danish University Hospital.

**Results:**

Six steps were included in the translation and cultural adaption. Discrepancies were addressed and revised by the expert committee until a consensus was reached on a reconciled version.

**Conclusion:**

As person-centred practice is a recognized standard of quality influencing the experience of care for healthcare professionals, service users, families and care partners, it has been important to translate the questionnaires PCPI-S, a measure of staff's perception of person-centred practice, and PCPI-C, a measure of patients’ perception of person-centred practice into Danish. Based on this, we now have a Danish instrument that may give the patients a voice by examining to what extent they experience person-centred care in our hospital. This will hopefully support learning and further development of a person-centred culture.

## Background

The development of person-centred cultures has become a global movement in healthcare that underpins many Western healthcare policy positions and strategic developments (1). Person-centred cultures prioritize the human experience and place compassion, dignity and humanistic caring principles at the centre of planning and decision-making and are translated through relationships that are built on effective interpersonal processes and where the core value of ‘respect for the person’ is paramount. The concept of person-centredness extends beyond mere individual treatment; it embodies a holistic understanding of individuals within their social contexts. In healthcare, person-centred care prioritizes patients’ preferences, needs and values, ensuring that they are active participants in their own care decisions. This approach has been linked to improved health outcomes, patient satisfaction and overall quality of care. Person-centredness is not a unidirectional activity focusing on ensuring that patients have a good care experience at the expense of staff wellbeing. So, whilst many organizations might focus on providing person-centred care, McCormack and McCance (2) articulate the importance of the broader idea of ‘person-centred practice’ where the focus is on creating cultures that enhance the wellbeing of all persons (including staff). Over the years, numerous frameworks and models have been developed to operationalize person-centred practices across various disciplines, reinforcing its significance as a guiding principle for effective and compassionate service delivery. As the landscape of care continues to evolve, the principles of person-centredness remain integral to fostering respectful and responsive care environments. McCormack and McCance define person-centred practice in healthcare as:

….an approach to practice established through the formation and fostering of healthful relationships between all care providers, service users and others significant to them in their lives. It is underpinned by values of respect for persons, individual right to self-determination, mutual respect and understanding. It is enabled by cultures of empowerment that foster continuous approaches to practice development. (2)

Internationally, person-centred practice is a recognized standard of quality care influencing the experience of care for healthcare professionals, service users, families and care partners. One challenge regarding developing person-centred cultures is that there is no universally accepted definition. According to de Salvi (2014), person-centred care is a philosophy that sees patients as equal partners in planning, developing and accessing care to make sure it is most appropriate for their needs (33). Different terms such as ‘person-centred’, ‘patient-centred', ‘family-centred’, individualized and personalized have been used as subcomponents to unfold person-centred care but often without being defined precisely [33; p. 8]. A systematic review of 60 articles explored the core elements of person-centred care in the health policy, medical and nursing literature, and three core elements were identified: patient participation and involvement, the relationship between the patient and the healthcare professionals and the context where care is delivered [33; p. 9].

In a newly established university hospital in Denmark, the overall vision in the area of nursing (valid from 2020 to 2025) is to place the beliefs and values of service users/patients at the centre of decision-making and thus recommends a person-centred approach to the development of evidence-based practice cultures (4). Thus, several departments decided to implement a person-centred approach guided by ‘The Person-Centred Practice Framework’ (PCPF) developed by McCormack and McCance (5).

The internationally recognized theoretical framework for person-centeredness provides a detailed exposition of its dimensions and offers guidance on how to implement these dimensions effectively in practice. At its core, the framework emphasizes the importance of establishing a therapeutic relationship between healthcare professionals and individuals, which includes families and care partners. It also emphasizes the importance of staff wellbeing. These relationships are built upon fundamental values such as respect for the individual, the right to self-determination and mutual respect and understanding (5).

**Figure 1:**
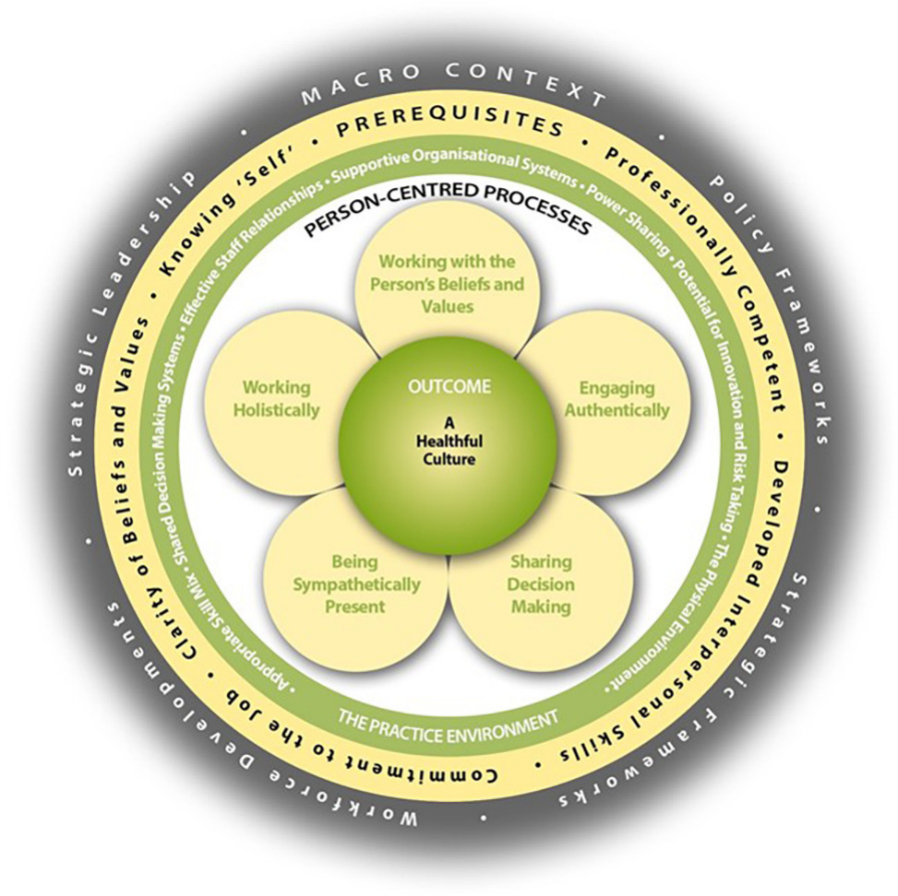
Person-Centred Practice Framework (5).

The framework is structured around four key domains, including prerequisites, the care environment, person-centred processes and person-centred outcomes, as shown in Figure 1. These domains are positioned within the broader macro context of the healthcare setting, the fifth dimension. The framework asserts that understanding and developing the attributes of healthcare staff are critical prerequisites for effectively managing the care environment. This management, in turn, enables the delivery of effective care through person-centred processes. Ultimately, this sequence is designed to lead to the achievement of person-centred outcomes, with the overarching goal being the creation and maintenance of a healthful culture.

The framework has been described as a practical approach to operationalizing person-centredness, acknowledging that whilst the concept is well understood in principle, its application in everyday practice remains challenging. Within the network dedicated to the consolidation of the PCPF, there is an increasing recognition of the complexities involved in implementing person-centred practices (6). These challenges are particularly pronounced for teams working within intricate and multifaceted organizational systems, where adapting the framework to suit specific contexts can be difficult (7). Thus, the framework not only serves as a guide but also highlights the need for continuous adaptation and reflection to effectively integrate person-centred practices into the fabric of healthcare delivery. Such challenges may influence how healthcare professionals can implement the framework into their practice and thereby live out the values of person-centred care.

The use of a framework such as that of the PCPF offers a systematic approach to decision-making in the development of person-centred cultures, and capturing the perspectives of the implementation process and experiences from both professionals and patients is important (8). The instruments Person-Centred Practice Inventory-Staff (PCPI-S) and Person-Centred Practice Inventory-Care (PCPI-C) questionnaires conceptually align with the four key domains and constructs in the Person-Centred Practice Framework (8, 9). Others, such as Vareta et al. (10), have used the PCPI instruments to provide evidence that would inform a starting point for defining strategies to move practice towards person-centredness and for monitoring changes (10). Tiainen et al. (11) showed that newly graduated or less experienced nursing professionals need support to explore person-centredness in their work, thus correlating the length of experience with the ability to provide person-centred care.

A research group at the newly established university hospital in Demark translated the Person-Centred Practice Framework (PCPF) into Danish (4), as well as the two associated instruments PCPI-S and PCPI-C used to measure outcomes, as a first step to implementing the PCPF at the hospital. In this paper, we report the process of translation and face validation of the instruments. Cross-cultural research can be conducted to explore the same questions in several cultures or measure differences across cultures (12).

### The Person-Centred Practice Inventory questionnaires

To measure the experience of person-centred practice from the perspectives of both caregivers and patients, two instruments were developed for all healthcare settings. Both instruments align with the theoretical domains of McCormack and McCance's Person-Centred Practice Framework and enable the measurement of the contextual and cultural issues that reflect the development of a healthful workplace culture (5, 13). The constructs within the Person-Centred Practice Framework are illustrated in Table 1. The instruments are developed in English, and both have been translated and structurally validated to French (14) whilst the PCPI-S also has been translated and culturally adapted to Norwegian, German, Spanish, Portuguese and Malaysian (15–20). The many culturally adapted instruments make it possible to compare the experiences of person-centred practices around the world. A Danish translation will complement the collection of validated questionnaires that document the development of person-centred practice.

**Table 1:** The questionnaires PCPI-S and PCPI-C domains and constructs in the Person-Centred Practice Framework.

Domains and constructs of the Person-Centred Practice Framework	PCPI-S questions	PCPI-C questions
The prerequisites of the Person-Centred Practice Framework		
Professionally competent	Q1–Q4	
Developed interpersonal skills	Q5–Q7	
Being committed to job	Q8–Q12	
Knowing self	Q13–Q15	
Clarity of beliefs and values	Q16–Q18	
The care environment of the Person-Centred Practice Framework		
Skill mix	Q19–Q21	
Shared decision-making systems	Q22–QQ25	
Effective staff relationships	Q26–Q28	
Power sharing	Q29–Q32	
Potential for innovation and risk-taking	Q33–Q35	
The physical environment	Q36–Q38	
Supportive organizational systems	Q39–Q43	
The care processes of the Person-Centred Practice Framework		
Working with patients’ beliefs and values	Q44–Q47	Q1–Q12–Q7–Q6
Shared decision-making	Q48–Q50	Q3–Q15–Q18–Q10
Engagement	Q51–Q53	Q11–Q16–Q9
Having sympathetic presence	Q54–Q56	Q14–Q5–Q2
Providing holistic care	Q57–Q59	Q13–Q8–Q4–Q17

#### Person-Centred Practice Inventory-Staff

The PCPI-S was developed to measure the experience of person-centred practice from the perspective of caregivers, and items were derived from a consensus-based process with experts on person-centredness described by Slater et al. (8). It consists of 59 items covering all constructs in the five domains of the Person-Centred Practice Framework. Each item is presented as a statement and scored on a 5-point-Likert scale ranging from strongly disagree, disagree, neutral, agree to strongly agree. The instrument has been tested for face validity and is psychometrically valid (8).

#### Person-Centred Practice Inventory-Care

The PCPI-C measures the experience of person-centred care from the perspective of care receivers/patients (9). The PCPI-C consists of 18 items designed as statements covering the construct of the ‘care processes’ domain of the Person-Centred Practice Framework. Each item is presented as a statement and scored on a 5-point-Likert scale ranging from strongly disagree, disagree, neutral, agree to strongly agree. It has been tested for face validity, and it is a psychometrically valid instrument (9).

### Aim

This paper aims to describe the translation into Danish and cultural adaption of the questionnaires PCPI-S, a measure of staff's perception of person-centred practice, and PCPI-C, a measure of patients' perception of person-centred practice.

## Method

**Figure 2:**
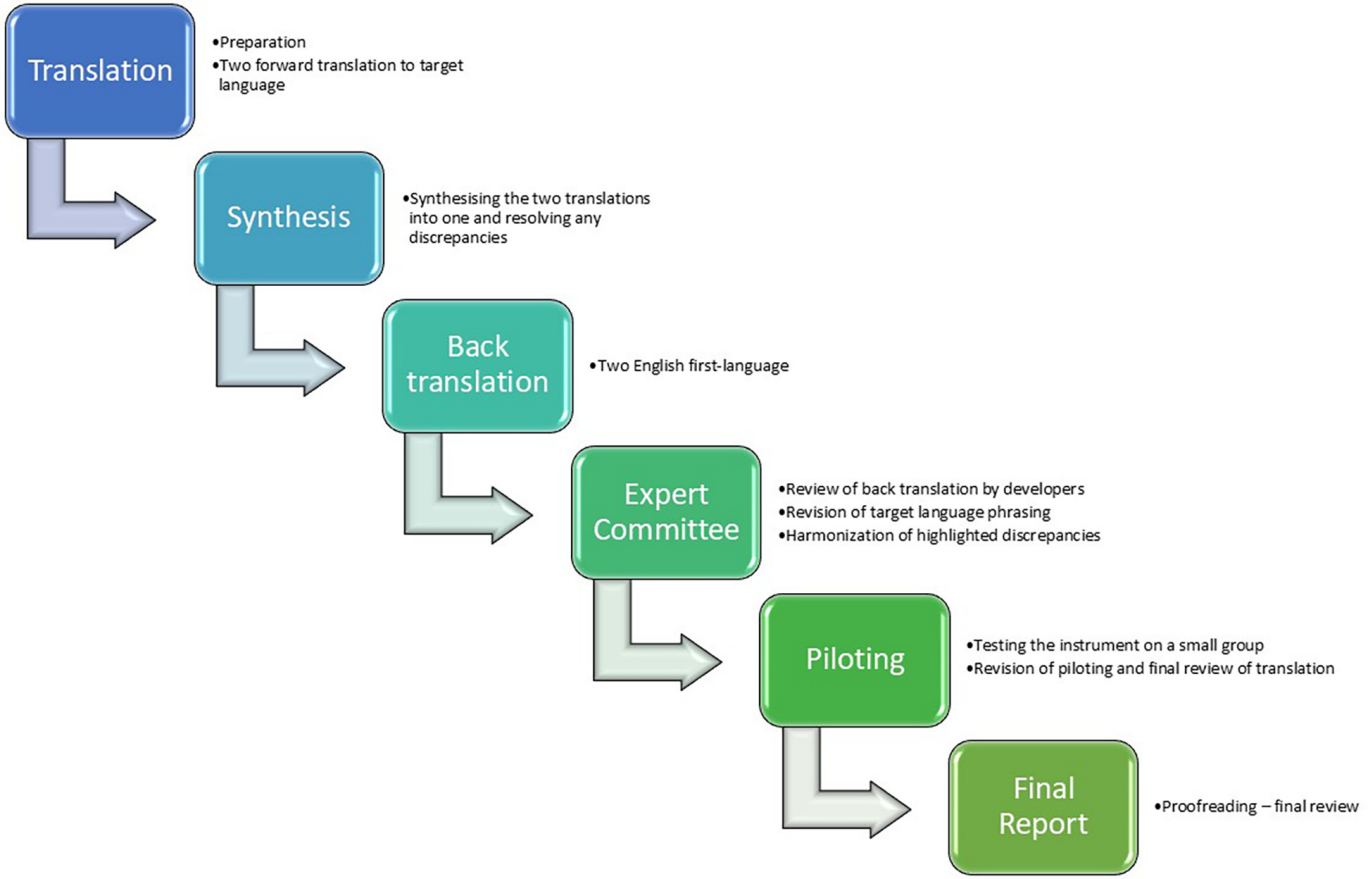
The steps of the translation process in a six-stage model.

To translate the PCPI-S and PCPI-C, back translation and cultural adaption methods were used (21, 22). The research group found that only using a forward–backward translation would not be sufficient to capture the complexity of the questions related to the theory of person-centred practice as developed by McCance and McCormack (23, 24). A more profound approach was needed, including a focus on implicit content and cultural adaption. The process was inspired by the principles of classic practice methods in translation and cultural adaption, as laid out by Ortiz-Gutiérrez and Cruz-Avelar (21, 25). At each step of the cultural adaption process, we collected evidence to support the equivalence between the original and the translated version. According to the recommendations, the following roles took part in the process: project manager, two bilingual translators educated as English-language correspondents (one translator had an in-depth understanding of the concept of person-centred practice), three Danish senior researchers, one professor and two English-speaking professors who were part of the development of the instruments. The translation and cultural adaption took place from September 2021 to March 2023 and was conducted within the context of a Danish University Hospital. Six steps were included in the translation process as illustrated in Figure 2. The study was approved by the Danish Data Protection Agency (REG-001-2023). According to Danish law, ethical approval is not required for non-invasive studies, including interview studies.

The steps of the translation process included the following:

• Translation
  ○ Preparation—initial work carried out before the translation work begins.
  ○ Forward translation—translation of the source versions of the questionnaires to Danish by two native-speaking translators.
• Synthesis
  ○ Synthesis—comparing and merging two forward translations into a single forward translation.
• Back translation
  ○ Back translation—translation of the new Danish language version back into two English versions by two independent translators.
• Expert committee
  ○ Review of back translations—comparison of the back-translated English versions of the instrument with the original version. Discussions with developers of the original tool to highlight and investigate discrepancies, which are then revised in the process of resolving the issues.
  ○ Revision of the target language phrasing.
  ○ Harmonization—to achieve a consistent approach to translation problems.
• Piloting
  ○ Piloting—testing the instrument on a small group of relevant patients and healthcare professionals to test alternative wording and to check the understandability, interpretation and cultural relevance of the translation.
  ○ Review of piloting and completion by the expert committee (26)—comparison of the patients’ and healthcare professionals’ interpretation of the translation to Danish with the original English version to highlight and amend discrepancies.
• Final report
  ○ Proofreading—final review of the translation to Danish to highlight and correct any typographic, grammatical or other errors carried out by one of the researchers. Report written at the end of the process documenting the development of each translation.

## Results

The translation and cultural adaption were performed according to the recommendations presented by Ortiz-Gutiérrez and Cruz-Avelar (25). The work carried out throughout the recommended steps is now described in detail.

### Translation

#### Preparation

Initial contact with the developers of the PCPI-S and PCPI-C was made, and permission for translation from English into Danish language was obtained. The measurement properties of the original tools were evaluated, and it was assessed that it was reasonable to use a five-step scale to measure person-centredness in a Danish clinical setting. The three Danish senior researchers and the professor agreed that the construct of the PCSI-S and PCSI-C measured culturally similar populations in the development and the target populations in which the adapted version was to be used. The developers confirmed that there were no ambiguities between the two populations and equivalence of concepts. The group assessed the feasibility of the process and agreed on a plan. The instrument developers agreed to be involved in the process.

#### Forward translation

Two bilingual and native speakers of the target language (Danish) independently translated the tools from English to Danish, thus creating two versions of both the PCSI-S and PCSI-C in the target language.

### Synthesis

Synthesis focused on comparing and merging two forward translations into a single forward translation. The three Danish senior researchers, the professor and the two forward translators compared the translations of both the PCSI-S and PCSI-C, discussed them against the English versions and agreed on a reconciled first version of both the PCSI-S and PCSI-C in the target language (Danish).

### Back translation

Two native English speakers who also had adequate knowledge of Danish back-translated the first drafts of the Danish version of PCSI-S and PCSI-C into English. The translators were uninformed about the final use of the translations, and new versions of the tools were created in the original language (English). To maintain the concepts of the PCSI-S and PCSI-C, the translations focused on a conceptual translation rather than a more literal back translation. The three Danish senior researchers and the professor discussed and agreed on discrepancies and then merged the two versions into a new English version that was sent to the developers.

### Expert committee review

To achieve cross-cultural equivalence, an expert committee was established consisting of the project manager, bilingual translators, three Danish senior researchers, one professor and two English-speaking professors who were part of the development of the instruments. According to Cruchinho et al. (26), this approach is also referred to as *harmonization* (see Figure 2).

#### Review of back translation

To ensure that the same meaning can be deduced from the new English versions and the original versions of PCSI-S and PCSI-C after the translation is converted to the original language, both versions were assessed by the developers. They pointed to three ambiguities between the original and back-translated English versions. One was simple spelling as they found typos in five questions (PCSI-S questions 36 and 59; PCPI-C questions 7, 9 and 20); one was in relation to the English wording or meaning in the back translation that differed from the original versions (PCSI-S questions 28, 33 and 57) and one was in relation to the conceptual equivalence of the translation. In the PCSI-S question 28, we changed the phrase ‘effective relations’ to ‘good relations’ as ‘effective relations’ has a different meaning in Danish. The typos and the wording were corrected and approved by the developers through e-mail correspondence. The differences in the translations were addressed and discussed first by e-mail with both developers and secondly by the expert group and one of the developers in person. The main conceptual ambiguity was in relation to the concept ‘patient’. The developers avoided the term ‘patient’ as it is not a person-centred phrase, and not all service users are patients. One developer commented on the use of the term ‘user’:

I have a comment on the PCPI-S and the “user/service user” issue. I completely understand the challenges you faced with this and indeed the term “service user” is also challenged here in the UK now and we have also discussed our own challenges with the term as language evolves in our health systems. I struggle with the term “user” as that term has such negative connotations in the English language. I also note that in a couple of items/questions you have used the term “patient”. So, I am wondering if the best thing at this point is to use the term “patient” throughout?.

In response to this, the other developer commented:

I agree that this is very challenging in terms of language. I realise there can be limitations in the wider context with using the term “patient”, but it would be my preference over “user”.

Taking the developers’ comments into account, the review group agreed that the terms ‘care recipient’, ‘service user’ or simply ‘user’ would not be understood appropriately in a Danish setting, and thus the term ‘patient’ was retained.

#### Revision of the target language phrasing

Based on the back translation review and the comments from the developers, discrepancies in the Danish version were discussed in the expert committee until a consensus was reached. Based on this version, a revised English version was created by the translator and sent to the developers. The developers accepted this version.

### Piloting

#### Piloting—testing the instrument

The final stage of the adaption was the pretest where the instrument was tested on a small group of relevant patients and healthcare professionals in order to test alternative wording and to check the understandability, interpretation and cultural relevance of the translation. The PCPI-S was tested among 10 nurses from a target setting, who completed the questionnaire followed by an interview to uncover what they thought was meant by each question and the chosen response. In question 28, the wording was adjusted, and the revised question was assessed by all 10 nurses to make sure the meaning was the intention. The PCPI-C was tested among 30 patients also from a target setting. They completed the questionnaire and were interviewed afterwards. This revealed that questions 4, 7 and 17 needed revisions to make sure they were understood as intended in the English version. The questions were revised and tested again among 30 patients.

#### Review of piloting and completion

The review group went through the corrected wording to check the understanding, interpretation and cultural relevance of the translation. Only a few grammatical revisions were made.

### Final report

#### Proofreading

To correct typographic, grammatical or other errors, the final versions of the instrument were proofread by the project manager.

A detailed report describing the translation process of actions taken in each step was written. We highlighted how the tasks were approached and how possible discrepancies were detected. We explained changes made and how quality was monitored to produce the cultural adaption.

## Discussion

This paper aims to describe the Danish translation and cultural adaption of the PCPI-S, a measure of the perception of staff's person-centred practice, and PCPI-C, a measure of patients' perceptions of person-centred practice. Translating an instrument into a second language is not a linear process of merely finding the exact, corresponding word. There are inherent risks with translation, because it may mean that parts of the original instrument are subtly altered, resulting in a version which measures something else than the original (27). Moreover, cross-cultural validation of an instrument is a complex and time-consuming process. Nevertheless, it is important to systematically document the method used to clarify specific risks of bias that could affect the research process and results (16, 26).

Many guidelines exist for translating and culturally adapting instruments (21, 26). However, as the goal of the study has been to achieve equivalence between two languages, we have chosen a model that is well described and builds on the classic method of translation, back translation and using an expert committee as key points to discuss the potential identified discrepancies in translations (25). The chosen approach ensures that a translated measurement tool uses language in the way it is understood culturally that is different from the original setting, yet does not lose its measurement properties (25–27). The benefit of traditional back translation is the possibility of holding the original language as a desired standard and as part of the translation process compared with the translated text with the objective of ensuring the interpretation is as close to the original language as possible (28). Back translation alone, however, may introduce false discrepancies and hence lead to inefficient use of time and effort due to the risk of mainly focusing on semantic equivalence—e.g., ensuring that the translation of items semantically matches the items in the ‘original version’ and not conceptual equivalence (29).

Herdman et al. (29) describe conceptual equivalence as the type of equivalence that verifies which domains, and their inter-relations, are important in the ‘target culture’ (e.g., the language being translated into) for the concept of interest evaluated by the instrument. In this study, the core concept of interest is ‘person-centred practice’ (PCP), as it is the concept that is measured using respectively PCPI-S and PCPI-C instruments. The PCP concept is well described in English (1) as well as in a Danish article (4). However, there may still exist a lack of clarity on how to use and understand the concept, especially in the target culture. For instance, the current study pinpoints how the English word *person*—as it is used in person-centred practice—during the translation process turned out to be difficult to translate into a Danish culture, as the Danish word *person* is unusual to be used in a health-related connection. The terms *patient* and *user* are more often used, but the review committee was unsure if the two words sufficiently covered the perception of the chosen English word *person*. An expert committee was used as part of the cultural validation, in which two developers of the original English version of the measurement took part. This opened a unique opportunity to discuss the conceptual unclarity of the choice of the most appropriate Danish terms. According to the translation and cultural adaption group (22), the inclusion of the instrument developers is one of the most important components of the cross-cultural adaption process, but one that most of the existing guidelines have not specifically addressed. The statement underscores, why we in the current study have placed great emphasis on this part of the cross-cultural adaption process.

After obtaining consensus among all experts, including two bilingual linguistics who ensured idiomatic and semantic equivalence, a pilot testing—similar to pretesting—was conducted. This involved the testing of the two measurements as recommended on a small number of healthcare professionals (PCPI-S) and patients (PCPI-C) (25, 30) (see Figure 1). Carrying out a pretest provides the identification of problems that may affect the reliability and validity of the translated version of the measurements, namely, related to the clarity and relevance of the core items, which in this context is the PCP concept. Furthermore, the pretesting gives the researchers the opportunity to consult the documentation of the previous steps and, if needed, to exclude semantic equivalence problems to replace or eliminate items from the measurements (26). Regarding the current study, only minor semantic equivalence problems were identified and subsequently revised by the expert committee. The Danish translations of the PCPI-S and the PCPI-C have been used to evaluate an action research study focusing on the development of a person-centred culture in a university hospital (31). The questionnaires were well received by both patients and nurses and results show that both patients and nurses experience care as person-centred (31).

### Limitations

Based on 42 guidelines on translation, adaption or cross-cultural validation of measurement instruments, Crunchinho et al. (26) suggest that the data obtained during the pretest can be submitted to a statistical analysis regarding the consistency and accuracy of the degree of agreement between reviewers. One opportunity is using a content validity index (CVI) to identify the content validity of the adapted version of the measurement. CVI is suitable for dichotomous answers but can also be used for Likert-type multiple-choice response formats by recoding the answers. Polit et al. (32) describe how using CVI instead of alternative indexes has advantages with regard to ease of computation, understandability, focus on agreement of relevance rather than consistency, and provision of both item and scale information. At the same time, it is from more sources underscored that using CVI may cause failure to adjust for chance agreement (32)—e.g., an issue of concern in evaluating indexes of inter-rater agreement, why the researchers should ensure that such procedures do not compromise the construct coverage of the original instrument. Based on that criticism, the researchers decided not to apply the use of CVI in the current study and instead highlight the use of the expert committee—including the two developers of the original version of the instruments (26). This ensured that the Danish translations were semantically consistent with the original questionnaires. In addition, the Norwegian language is closely related to Danish and a Norwegian study by Bing-Jonsson et al. (16) performed a psychometric evaluation comparing the Norwegian version with the original version of PCPI-S and found that the psychometric properties were acceptable (16).

## Conclusion

As person-centred practice is a recognized standard of quality influencing the experience of care for healthcare professionals, service users, families and care partners, it has been important to conduct the translation into Danish and cultural adapt the questionnaires PCPI-S, a measure of staff's perception of person-centred practice, and PCPI-C, a measure of patients' perception of person-centred practice. Using an internationally accepted approach to translation and cultural adaption, and between the original and back-translated English versions, several ambiguities were found. The main conceptual ambiguity was related to the concept ‘patient’. An Expert Committee consisting of the researchers and two developers of the original English version discussed the discrepancies and conducted a harmonization process, followed by a pilot testing of the translated instrument. The pilot testing highlighted other ambiguities, which were discussed and revised by the expert committee. The revised Danish version was retested. Only a few grammatical revisions were made, and a detailed report describing the translation process of actions taken in each step completed the translation and cultural adaption process. Based on this, we now have a Danish instrument that gives the patients a voice by examining to what extent they experience person-centred care in our hospital. This will hopefully support learning and further development of a person-centred culture.

## Data Availability

The raw data supporting the conclusions of this article will be made available by the authors, without undue reservation.
