# Correction: Danish translation and cultural adaption of the Person-Centred Practice Inventory-Staff and Person-Centred Practice Inventory-Care Questionnaires

**DOI:** 10.3389/frhs.2025.1663804

**Published:** 2025-07-28

**Authors:** Elizabeth Rosted, Mette Kjerholt, Bibi Hølge-Hazelton, Tanya McCance, Brendan McCormack, Thora Thomsen

**Affiliations:** ^1^ Department of Oncology and Palliative Care, Zealand University Hospital, Roskilde, Denmark; ^2^ Department of Regional Health Research, University of Southern Denmark, Odense, Denmark; ^3^ Department of Hematology, Zealand University Hospital, Roskilde, Denmark; ^4^ Research Support Unit, Zealand University Hospital, Roskilde, Denmark; ^5^ Faculty of Life and Health Sciences, School of Nursing and Paramedic Science/Institute of Nursing and Health Research, Ulster University, Coleraine, United Kingdom; ^6^ Faculty of Medicine and Health, The University of Sydney, Sydney, NSW, Australia; ^7^ Department of Ear, Nose, Throat and Maxillofacial Surgery, Zealand University Hospital, Køge, Denmark

**Keywords:** translation, person-centred practice, cross-cultural adaptation, person-centred, measurement

Missing citation

Wrong

On page 2 line 161: The reference for [33] was erroneously written as [(33); p. 8]. It should be [33].

The original version of this article has been updated.

On page 2 line 165: The reference for [33] was erroneously written as [(3); p. 8]. It should be [33; p. 8].

The original version of this article has been updated.

On page 2 line 170: The reference for [33] was erroneously written as [(3); p. 213, (33); p. 9]. It should be [33; p. 9].

The original version of this article has been updated. [33] is de Silva, 2014.

